# ADHD in adolescents with borderline personality disorder

**DOI:** 10.1186/1471-244X-11-158

**Published:** 2011-09-30

**Authors:** Mario Speranza, Anne Revah-Levy, Samuele Cortese, Bruno Falissard, Alexandra Pham-Scottez, Maurice Corcos

**Affiliations:** 1 Centre Hospitalier de Versailles. Service de Pédopsychiatrie. Le Chesnay, France and EA40/47 UVSQ, France; 2 INSERM U669, Univ Paris-Sud and Univ Paris Descartes, UMR-S0669, Paris, France; 3 Centre de Soins Psychothérapeutiques de Transition pour Adolescents, Hôpital d'Argenteuil, F-95107, Argenteuil, France; 4 Institute for Pediatric Neuroscience, New York University Child Study Center. 215 Lexington Ave, 14th Floor. New York, NY 10016, USA; 5 Clinique des Maladies Mentales et de l'Encéphale, Hôpital Sainte-Anne, Paris, France; 6 Institut Mutualiste Montsouris, Département de Psychiatrie de l'Adolescent et du Jeune Adulte, Paris, France

## Abstract

**Background:**

The aims of this study were to assess the prevalence of a comorbid Attention Deficit Hyperactivity Disorder (ADHD) diagnosis in Borderline Personality Disorder (BPD), and its impact on the clinical presentation of BPD in adolescents, and to determine which type of impulsivity specifically characterizes adolescents with BPD-ADHD.

**Methods:**

ADHD diagnoses were sought in a sample of 85 DSM-IV BPD adolescents drawn from the EURNET BPD. Axis-I and -II disorders were determined with the K-SADS-PL and the SIDP-IV, respectively. Impulsivity was assessed with the BIS-11.

**Results:**

11% (N = 9) of BPD participants had a current ADHD diagnosis. BPD-ADHD adolescents showed higher prevalence of Disruptive disorders **(**Chi^2 ^= 9.09, p = 0.01) and a non-significant trend for a higher prevalence of other cluster B personality disorders (Chi^2 ^= 2.70, p = 0.08). Regression analyses revealed a significant association between Attentional/Cognitive impulsivity scores and ADHD (Wald Z = 6.69; p = 0.01; Exp(B) = 2.02, CI 95% 1.19-3.45).

**Conclusions:**

Comorbid ADHD influences the clinical presentation of adolescents with BPD and is associated with higher rates of disruptive disorders, with a trend towards a greater likelihood of cluster B personality disorders and with higher levels of impulsivity, especially of the attentional/cognitive type. A subgroup of BPD patients may exhibit developmentally driven impairments of the inhibitory system persisting since childhood. Specific interventions should be recommended for this subsample of BPD adolescents.

## Background

Borderline personality disorder (BPD) is an impairing mental disorder that concerns 1-2% of the general population. It is characterized by a pervasive pattern of instability in affect regulation, impulse control, interpersonal relationships, and self-image [1]. Although BPD is usually diagnosed in adults, symptoms of BPD can often be traced back to childhood [2]. Several studies have shown that specific features of BPD, such as self-harm, impulsivity and emotional dysregulation, present during childhood or adolescence, are predictive of BPD diagnoses in adulthood [3-5]. Among these, impulsivity in particular is regarded as a core feature of BPD [1,6]. Impulsivity is associated with factors contributing to the severity of the disorder, such as suicidal/self-harming behaviours or increased risk for substance abuse [7,8]. Impulsivity in BPD has been related to dysfunction in inhibitory systems mediated by fronto-striatal circuits [9-12].

Impulsivity, along with inattention and hyperactivity, is also one of the core symptoms of Attention-Deficit/Hyperactivity Disorder (ADHD)[13]. Impulsivity may contribute to motor (overactivity), cognitive (poor cognitive control), emotional (uncontrolled tempers) and interpersonal (social disinhibition) dysfunctions reported in patients with ADHD [14]. Meta-analytical reviews have confirmed that deficient inhibitory functions, especially executive motor inhibition, are among the most robust findings in ADHD research [15,16]. Response inhibition deficits in ADHD have been related to functional and volumetric changes in the right inferior frontal cortex (IFC) and in its associated circuitry involving projections from the basal ganglia and into the striatum [17,18].

Thus, ADHD and BPD share dysregulation in emotional and impulse control, with a possible mediating role of a dysfunction of neuronal inhibitory systems. Interestingly, several reports concerning the greater-than-chance co-occurrence of these two disorders have been published [19-21]. Since ADHD, as a neuro-developmental disorder, appears earlier than BPD, it has been suggested that ADHD may contribute to the development of BPD [22]. Longitudinal prospective studies indicate that adolescents and young adults with a childhood history of ADHD are more likely than those without that history to have a personality disorder, with a higher risk for borderline and antisocial personality disorders than for others [23,24]. Stepp and colleagues recently published the first longitudinal study to examine ADHD and ODD symptom trajectories as specific childhood precursors of BPD symptoms in adolescent girls [25]. They performed a series of latent growth curve models on two cohorts of girls annually assessed between the age of 8 and 14. They found that higher levels of ADHD and ODD scores at age 8 uniquely predicted BPD symptoms at age 14; over and above depression symptoms at outcome. However, as suggested by Davids and Gastpar [26], BPD subjects are likely to be an heterogeneous group, with some subjects characterized by prominent impulsive features, others by prominent affective or dissociative features. ADHD may thus represent a risk factor for BPD patients with a predominance of impulsivity features. However, impulsivity is not a unidimensional construct and authors regard it as composed of several dimensions, such as motor, attentional and cognitive impulsivity. Data from the literature report the existence of deficits in the three facets of impulsivity in ADHD subjects [27]. However, less is known about subjects with BPD and ADHD. As adolescence is a key period for the onset of personality disorders, focusing on BPD adolescents with persistent ADHD comorbidity since childhood can cast light on the developmental trajectory of BPD. As the literature concerning these topics is sparse, our study aimed to: 1) assess the past and current prevalence of a comorbid ADHD/BPD diagnosis and its impact on the clinical presentation of borderline personality disorder in adolescents and; 2) determine which type of impulsivity specifically characterizes adolescents with BPD-ADHD.

## Methods

### Participants

The study sample was drawn from a European research project investigating the phenomenology of BPD in adolescence (European Research Network on Borderline Personality Disorder, EURNET BPD)[28]. The research network was composed of five university psychiatric centers in France, Belgium, and Switzerland. During the period between January and December 2007, all consecutively admitted adolescent in and out-patients (aged 15 to 19) were clinically screened by the consulting psychiatrists to look for a diagnosis of BPD according to the DSM-IV criteria. Before the beginning of the project, the outline of the study had been presented to clinicians in research meetings and specific questions concerning the DSM-IV criteria for BDP diagnosis had been discussed. Clinicians had to fulfil a questionnaire specifying all BPD DSM-IV criteria before referring the participants to the research team. Exclusion criteria were a diagnosis of schizophrenia or any chronic and/or serious medical illness involving vital prognosis. Adolescents fulfilling the criteria for BPD according to clinicians were further investigated with a research protocol which consisted in a diagnostic evaluation of Axis-I and Axis-II disorders (with confirmation of the BPD diagnosis with the SIDP-IV interview) and a self-report questionnaire eliciting socio-demographic data and psychopathological features. For the present study, only participants with a confirmed diagnosis of BPD according to the SIDP-IV interview were included in the final sample.

### Diagnosis of BPD and ADHD

Diagnosis of BPD was ascertained through the Structured Interview for DSM-IV Personality (SIDP-IV), a semi-structured interview assessing each of the ten DSM-IV personality disorders, including BPD [29]. The reliability and validity of the SIDP-IV have been established in adolescents and young adults and have been validated in French [30-33]. The profile of borderline symptoms in the four domains of functioning that are potentially impaired in borderline patients (affects, cognition, impulsivity and interpersonal relationships), was assessed with the Revised Diagnostic Interview for Borderlines (DIB-R)[33,34].

ADHD diagnosis and other comorbid DSM-IV axis-I disorders were assessed using the Schedule for Affective Disorders and Schizophrenia for School-Age Children (K-SADS-PL), which is a semi-structured diagnostic interview designed to assess current and past episodes of psychopathology in children and adolescents according to DSM-IV criteria [35]. The interview begins with a screening interview for the primary symptoms of the different diagnoses of the DSM-III and IV. If the patient has clinical manifestations of the primary symptoms associated with the specific diagnosis, the appropriate supplements are administered. Regarding ADHD specifically, the screening interview includes 4 items exploring Inattention (difficulty sustaining attention on task or play activities; easily distracted), Hyperactivity (difficulty remaining seated) and Impulsivity (acting before thinking). Items are scored as absent (rating of 0), subclinically significant (rating of 1) or clinically significant (rating of 2). ADHD screening data were obtained for the entire sample. A complete assessment of all DSM-IV ADHD symptoms was performed only for adolescents with a score of at least 2 (threshold criterion) on either the current or past ratings of any of the four screening items, as recommended by the K-SADS-PL administration guidelines[36]. As impulsivity is a common criterion shared by both BPD and ADHD, there is the risk of overestimating ADHD diagnosis in BPD. To explore this potential bias, the diagnosis of ADHD was assessed twice, with and without the impulsivity criteria listed in the DSM-IV.

Diagnostic interviews were conducted by a research team of five doctoral or master's level clinicians (psychologists or psychiatrists) familiar with DSM-IV Axis-I/II disorders and trained in the assessment and treatment of adolescents with psychiatric disorders. To reach high levels of reliability, the research evaluation team participated in several training sessions, including commented scoring of videotaped interviews and a training session conducted by the developers of the K-SADS (Boris Birmaher, MD and Mary Kay Gill, MSN). Concerning BPD diagnosis. special attention was paid to the question of the one-year duration of the symptoms and to the pervasive and persistent nature of the traits, unlikely to be limited to episodes of an Axis-I disorder. Final diagnoses were established by the best-estimate method on the basis of the interviews and any additional relevant data from the clinical record according to the LEAD standard [37]. Inter-rater reliability for the SIDP-IV was calculated from independent ratings of ten videotaped interviews. The Kappa coefficient for the presence/absence of BPD was very good (0.84). The intraclass correlation coefficient for SIDP-IV borderline score was excellent (0.95). At the end of the clinical assessment session, an overall level of psychosocial functioning was calculated for each patient according to the Global Assessment of Functioning (GAF)[38].

### Self-assessment of psychopathology

Impulsive behaviors were investigated using the French validation of the Barratt Impulsiveness Scale (BIS-11)[39]. The BIS-11 is a widely used and well-validated personality measure of impulsivity. The structure of the instrument allows for the assessment of three components: Cognitive/Attentional impulsiveness (the ability to focus on the task at hand and the cognitive speed in decision making), Motor impulsiveness (acting without thinking and restlessness), and Non-Planning impulsiveness (lack of future-oriented problem-solving strategies). Finally, to explore the overall impact of ADHD diagnosis on the family functioning of borderline adolescents, participants completed the general functioning scale of the Family Assessment Device which is a well-established scale to assess family functioning [40].

### Statistical analysis

To explore a possible influence of ADHD on the clinical presentation of borderline personality disorder, BPD adolescents with (BPD-ADHD) and without (BPD) current comorbid ADHD disorder were compared for sociodemographic and clinical characteristics (Axis-I and Axis-II, BPD severity, impulsivity, family functioning and general functioning). To take into account the variability between centers, we used the Mantel-Haenszel chi-square statistic for categorical variables, preceded by the Breslow-Day test to assess the homogeneity of the odds ratios of the recruitment centres. For the continuous variables, we used the nested ANOVA statistic controlling for the recruitment centers. To reduce the number of statistical comparisons and comparisons with few observations, Axis-I and Axis-II diagnoses were included as groups of related disorders. Finally, to explore which type of impulsivity was specifically associated with BPD-ADHD adolescents, a logistic backward stepwise regression analysis was performed with presence or absence of ADHD as a dependent variable and with the three scores on the Barratt Impulsiveness Scale and the recruitment centers as independent variables. For all the analyses, the significance level was set at p = .05, 2-sided. Statistical analyses were performed using the 18th version of the Statistical Package for Social Sciences (SPSS Inc., Chicago, IL).

### Ethical statement

This study was approved by the ethics committee of the Hôtel Dieu Hospital in Paris (authorization n° 0611259). Results were collected in an anonymous database according to the requirements of the French national committee for private freedoms. All participants, adolescents and parents, signed informed consent after receiving a full description of the study, explanation of its purpose, and information about the confidentiality of the data.

## Results

One-hundred and seven adolescents with a DSM-IV clinical diagnosis of BPD were referred to the study by their psychiatrists. Of these subjects, 85 fulfilled SIDP-IV criteria for a BPD and composed the final sample of the study. There were no significant differences between the recruitment centres in terms of subject age and educational level, numbers of borderline criteria and in/outpatient ratio. The mean age of the sample was 16.3 ± 1.4 yrs; 74 (87%) were girls. The sample had a severe clinical profile: 67% (n = 89) of the subjects were recruited from inpatient units and had a mean score of 17.6 ± 3.9 on the SIDP-IV borderline diagnostic criteria (minimum required score being 15 with a maximum of 27). The most frequently endorsed criteria (more than 85% of the sample) were Impulsivity, Suicidal/self-mutilating behavior, Affective instability and Inappropriate anger. The majority of adolescents met the criteria for at least one Axis-I disorder (N = 76, 89%). Mood disorders were the most frequently observed comorbidity (N = 47, 55.3%) followed by eating disorders (N = 27, 31.8%), disruptive behavior disorders (N = 22, 25.9%), and substance use disorders (N = 17, 20%). The sample showed a severe impairment in the overall level of psychosocial functioning with a mean GAF score of 47.2 ± 14. 76% of the samples were currently under medication. Antidepressants and antipsychotics were the most commonly prescribed drugs. No patients were currently taking stimulants, although 2 patients had been under methylphenidate treatment during childhood.

### ADHD comorbidity and ADHD symptom profiles in BPD adolescents

Table 1 reports the frequency of current and past ADHD symptoms from the screening interview for the entire sample of borderline adolescents. Subclinical and clinical symptoms of Sustained attention, Distractibility, Motor hyperactivity and Impulsivity were evenly distributed across the sample. Among the 85 BPD adolescents, 21% (N = 18) showed at least one impairing, clinically significant, current or past ADHD symptom at the screening interview and were administered the K-SADS complete ADHD diagnostic supplement. 15% (N = 13) fulfilled the diagnostic criteria for a past ADHD diagnosis and 11% (N = 9) for a current ADHD diagnosis (with a diagnostic persistence of 69% between childhood and adolescence). All the current cases were of the combined type. There was no difference in the rates of ADHD according to the sex of BPD adolescents (Boys = 11.1% vs Girls = 10.5%, p = ns). Assessment of ADHD diagnosis without including DSM-IV impulsivity criteria did not result in any modification of current ADHD comorbidity rates. In just two participants with current ADHD, the diagnostic subtype shifted to purely inattentional forms.

**Table 1:** Current and past ADHD symptoms in BPD adolescents (N = 85)

	Current ADHD symptoms		Past ADHD symptoms	
		
ADHD symptoms*	Subclinical symptoms	Clinical symptoms	Subclinical symptoms	Clinical symptoms
	**N (%)**	**N (%)**	**N (%)**	**N (%)**

**Sustained attention**	10 (12)	6 (7)	8 (9)	10 (12)
**Distractibility**	11 (13)	8 (9)	14 (16)	7 (8)
**Motor hyperactivity**	11 (13)	9 (11)	11 (13)	8 (9)
**Impulsivity**	17 (20)	9 (11)	17 (20)	11 (15)
**At least 1 ADHD symptom**	27 (32)	12 (14)	28 (33)	18 21)
**- without Impulsivity**	22 (26)	10 (12)	24 (28)	12 (14)
**DSM-IV Diagnosis of ADHD**		9 (11)		13 (15)

* ADHD symptoms as assessed with the K-SADS-PL ADHD screening section. BPD = Borderline personality disorder; ADHD = Attention Deficit Hyperactivity Disorder.

### The influence of ADHD diagnosis on co-occurring Axis-I and Axis-II disorders

There were no significant differences in sociodemographic characteristics between borderline adolescents with and without a current ADHD diagnosis (Table 2). Borderline adolescents with a current ADHD diagnosis (BPD-ADHD) showed a higher prevalence of disruptive disorders compared to borderline adolescents without ADHD (BPD). The effect was uniform across the recruitment centres (Breslow-Day Chi^2 ^= 1.04, p = 0.79) and was mostly related to a higher prevalence of Oppositional defiant disorders in BPD-ADHD adolescents (Chi^2 ^= 3.75, p = 0.04). No other significant difference was found for the prevalence of Axis-I groups of related disorders. Axis-II clusters of personality disorders were evenly distributed between BPD-ADHD and ADHD adolescents, with only a non significant association between BPD-ADHD adolescents and the other personality disorders of the cluster B (24% vs 56%, Chi^2 ^= 2.7, p = 0.08)(Table 3).

**Table 2:** Sociodemographic characteristics of BPD adolescents with and without ADHD

	BPD (N = 76)		BPD-ADHD (N = 9)		Analysis *	
			
	N	%	N	%	Chi ^2^	p
**Age **(m ± sd) §	16.6	1.5	16.3	1.0	0.09	0.78
**Sex (**Females)(%)	66	87	8	89	0.01	0.93
**Educational level (%)**					0.06	0.81
< Secondary diploma	72	95	9	100		
≥ Secondary diploma	4	5	0	0		
**SES **(Father)(**%)**					0.18	0.73
Executive/Intellectual	40	51	5	50		
White collar/Manual	30	40	2	25		
No activity	6	9	2	25		
**Living with family **(%)	63	84	7	80	0.56	0.45
**Inpatient status **(%)	49	66	7	78	0.01	0.98

*Mantel-Haenszel chi-square statistic adjusted on recruitment centres. § Nested analysis of variance controlling for recruitment centres

**Table 3:** Prevalence of Axis-I and Axis-II disorders in BPD adolescents with and without comorbid ADHD

	BPD (N = 76)		BPD-ADHD (N = 9)		Analysis *	
			
Disorders	N	%	N	%	Chi ^2^	p
**Axis-I disorders**^#^						
Mood disorders	43	56.6	4	44.4	0.41	0.52
Anxiety disorders	20	26.3	2	22.2	0.03	0.86
Eating disorders	25	32.9	2	22.2	0.06	0.81
Disruptive behavior disorders	13	17.1	7	77.8	9.09	**0.01**
Substance related disorders	15	19.7	2	22.2	0.04	0.85
**Axis-II disorders^§^**						
Cluster A	8	10.5	0	0	0.47	0.49
Cluster B	18	23.7	5	55.6	2.70	0.08
Cluster C	41	53.9	4	44.4	0.02	0.88

# K-SADS; § SIDP-IV; * Mantel-Haenszel chi-square statistic controlling for recruitment centres.

Axis-I disorders: Mood disorders = Major depression, Dysthymia and Bipolar disorder. Anxiety disorders = General anxiety disorder and Post traumatic stress disorders. Eating disorders = Anorexia or Bulimia. Disruptive behavior disorders = Oppositional Defiant Disorder and Conduct disorder. Substance related disorders = Alcohol and drug related disorders.

Axis-II disorders: Cluster A disorders = Paranoid, Schizoid and Schizotypal personality disorders; Cluster B = Antisocial, Histrionic and Narcissistic personality disorders; Cluster C = Avoidant, Dependent and Obsessive-Compulsive personality disorders.

### The influence of ADHD diagnosis on BPD symptomatological profile and on psychopathological features

BPD-ADHD adolescents showed a different profile of borderline symptoms as assessed by the DIB compared to BPD adolescents. BPD adolescents scored higher in the domain of cognition, whereas BPD-ADHD scored higher in the domain of impulsivity. Moreover, borderline adolescents with ADHD showed higher scores on all measures of impulsivity as assessed by the Barratt Impulsiveness Scale, with differences reaching significance for the Attentional/Cognitive impulsivity subscale (F = 8.57, p = 0.01). The non significant results of the nested anova concerning the recruitment centres imply that is unlikely that the differences between BPD adolescents with and without ADHD could be explained by differences in the centres. BPD and BPD-ADHD adolescents showed a similar level of family dysfunction and a similar overall level of psychosocial functioning (Table 4). Finally, the logistic regression analysis revealed a significant positive association between Barratt's Attentional/Cognitive impulsivity and ADHD diagnosis in borderline adolescents (Wald Z = 6.69; p = 0.01; Exp(B) = 2.02, CI 95% 1.19-3.45) with no effects of the recruitment centers.

**Table 4:** The influence of ADHD diagnosis on borderline symptomatology and on impulsivity

	BPD (N = 76)		BPD-ADHD (N = 9)		Analysis *	
			
	M (SD)	95% CI	M (SD)	95% CI	F (p)§	F (p)#
**DIB-R**						
- Affect	1.6 (0.6)	1.5-1.8	1.2 (0.8)	0.6-1.9	2.82 (0.06)	2.01 0.07)
- Cognition	1.1 (0.8)	0.9-1.3	0.3 (0.5)	-0.5-0.7	6.32 (**0.02**)	1.24 0.29)
- Impulsivity	1.5 (0.6)	1.3-1.7	1.9 (0.3)	1.6-2.5	4.10 (**0.04**)	0.34 0.89)
- Interpersonal relationships	1.3 (0.8)	1.1-1.4	1.4 (0.7)	0.1-2.0	0.04 (0.84)	3.59 (0.05)
**Barratt Impulsiveness Scale (BIS-11)**						
- Attentional/Cognitive impulsivity	12.2 (3.1)	11.3-13.0	17.6 (2.9)	14.0-21.2	8.57 (**0.01**)	0.48 0.82)
- Motor impulsivity	11.9 (4.7)	10.6-13.0	17.6 (3.6)	13.2-22.0	1.99 (0.17)	1.24 0.30)
- Non-planning impulsivity	16.5 (5.8)	14.7 (18.1)	24.2 (1.9)	21.8-26.6	3.57 (0.07)	1.42 (0.22)
**Family Assessment Device**	16.0 (7.8)	14-18	22.4 (8.6)	12-33	1.48 (0.23)	1.21 (0.31)
**Global Assessment of Functioning**	47.1 (14.4)	44-50	47.6 (12.4)	38-57	0.24 (0.63)	0.88 (0.52)

* Nested analysis of variance controlling for recruitment centres. § Differences between diagnostic groups. # Differences between recruitment centres (nested within diagnostic groups)

## Discussion

The aim of this study was to explore the prevalence of a comorbid ADHD-BPD diagnosis and its impact on the clinical presentation of borderline personality disorder adolescents, and to explore which type of impulsivity is specifically associated with BPD-ADHD adolescents. To our knowledge, this is the first study investigating ADHD in BPD in this specific age group.

Concerning the prevalence of ADHD diagnosis in our sample, we found a current rate of 11%. This result is close to the 16% rate found by Philipsen and colleagues in a sample of adult BPD female patients [19], notwithstanding some methodological differences between the studies. In the Philipsen's study, current ADHD was diagnosed by self-assessment using the short version of the WURS (for childhood ADHD symptoms) and the adult ADHD-Checklist, whereas in our study diagnosis was ascertained by experienced clinicians using a valid and reliable diagnostic interview integrating all relevant data from the clinical records of the patients, including parental reports. Although the current prevalence observed here may appear not very high, up to 46% of the subjects presented at least one symptom with a clinical or subclinical significance and some impact on functioning in the ADHD screening, eventually qualifying for a diagnosis of ADHD-NOS. It is interesting to note that symptoms of inattention, hyperactivity and impulsivity were evenly distributed across the sample. This points to the fact that all types of ADHD symptoms, not solely impulsivity, are frequently found in BPD adolescents. Moreover, comorbidity rates did not change when diagnosis was made without including impulsivity, thus reducing the criticism of an overestimation of ADHD diagnosis in BPD due to symptom overlap.

The results of this study also show that the presence of a comorbid ADHD diagnosis influences the clinical presentation of BPD in adolescents. ADHD in BPD was significantly associated with a greater likelihood of disruptive disorders (particularly ODD) and with a trend for a greater likelihood of other cluster B personality disorders (histrionic, narcissistic and antisocial personality disorders). This result is not surprising since in longitudinal studies, ODD in childhood as well as antisocial behaviours in adolescence and adulthood have been frequently observed as main outcomes for ADHD children [41,42]. Impulsivity has been suggested as an important mediator of this negative outcome among ADHD children [43,44]. The role played by impulsivity in the relationship between ADHD and outcome was indirectly suggested in our study by the observation of higher levels of impulsivity on all Barratt subscales (although significant only for the Attentional/Cognitive subscale) and in the specific domain of impulsivity on the DIB-R in the BPD-ADHD group. The impulsivity dimension of the DIB-R includes several externalizing behaviours, driven by impulsivity, such as substance abuse, promiscuous sex, reckless driving or self-harming/suicidal behaviours. A reverse tendency on the DIB-R was observed in the domain of cognition, with borderline adolescents without ADHD showing a clinical profile characterized by more internalising symptoms such as odd thinking, unusual perceptual experiences or paranoid/quasi-psychotic experiences. This dual dissociation on the DIB-R indices between BPD and BPD-ADHD adolescents moderates the conclusions reached by Philipsen and colleagues [19], suggesting that this association might not be equivalent to a more severe form of the borderline disorder, but could correspond to a specific subtype of BPD with high impulsivity associated with an ADHD profile. This hypothesis is in line with recent conclusions drawn by Ferrer and colleagues [21] who have suggested that BPD patients should be distinguished in two subgroups according to the presence or absence of ADHD, with the former subgroup showing a specific profile of impulsive comorbidity. Moreover, these results recall the ICD-10 conceptualization of the emotionally unstable personality disorders, which specifically includes an impulsive sub-type alongside the typical borderline profile [45]. Our study suggests that the ICD-10 impulsive sub-type could be more developmentally driven, with ADHD symptoms persisting since childhood. This proposal could be of interest for the possible inclusion of a developmental perspective in the DSM classification of personality disorders. A similar proposal for differentiating borderline patients according to specific developmental features has already been suggested by Andrulonis [46] who, in a sample of DSM-III BPD adults, identified a separate group of patients showing severe hyperactivity, distractibility and/or learning disabilities and episodes of behavioral dyscontrol. This group reported hyperactive and aggressive behaviours during childhood and antisocial acting-out with drug/alcohol abuse during adolescence but, like our sample, did not show any micro-psychotic episodes. This association also supports one of the developmental routes to BPD suggested by Nigg [12], which he has termed as the primary impulsivity route, as opposed to the traumatogenic route more related to severe disruptions in early caregiving experiences and mainly affecting the development of affect regulation. For this author, this impulsive BPD subgroup could arise from weak executive response inhibition mechanisms, leading to extremes of impulsivity, behavioural disturbances during childhood, inappropriate interpersonal relations, and a cascade of negative socialization experiences leading to personality disturbances. From a temperamental perspective, specific features related to impulsivity in ADHD children, such as Novelty Seeking, have also been found to increase the risk of development of BPD in adulthood [47]. Data supporting this theoretical perspective have been reported by Lampe and colleagues [48] who assessed various motor and cognitive inhibitory functions in adult ADHD patients, with and without BPD, compared to subjects with BPD alone and controls. In this study, ADHD subjects (whether or not comorbid with BPD) had higher scores than BPD subjects on all behavioural subscales of the BIS and showed impaired inhibition on the Attentional Network Task (Stop and Interference). Conversely, BPD subjects (without comorbid ADHD) did not differ from their matched controls, a result which led the authors to conclude that an impairment of inhibitory control could be a core deficit of BPD only when associated with ADHD. This result suggests that the cognitive component of inhibitory control may play a specific role in the phenomenology of the impulsive/developmental sub-type of BPD. Results from the regression analysis of our study showed a specific association between Barratt's Attentional/Cognitive Impulsiveness and ADHD diagnosis in borderline adolescents. The Attentional/Cognitive impulsivity of the BIS-11 involves several clinical features in the domain of attention and of cognitive stability: the inability to inhibit irrelevant information held in working memory and to focus on the task at hand leading to distractibility [49]; and an excessive cognitive speed in decision-making [50] with an aversion to externally imposed delays [51] leading to cognitive and behavioural mistakes or acting-out behaviours, especially under emotional conditions [52]. Attentional impulsivity has been linked to the the dorsolateral prefrontal cortex [53] whereas cognitive impulsivity has been correlated to the orbitofrontal/ventromedial areas of the prefrontal cortex, especially the more anterior sector of this region, the frontal pole [49]. Some preliminary results support the hypothesis that orbitofrontal/ventromedial prefrontal dysfunction may underlie some of the behavioural manifestations of BPD-ADHD patients [54,55], but more data are needed, especially in adolescent samples.

Some limitations of the current study must be taken into consideration when interpreting the findings.

First, the main limit of the study is its cross-sectional design with data on childhood ADHD diagnosis collected retrospectively. Only longitudinal studies can directly support the identification of the developmental pathways leading from childhood to adult psychopathology. This is even more important if we consider that these diagnostic constructs tend to overlap, particularly in the realm of impulsivity. However cross-sectional studies on comorbid disorders in specific populations, such as adolescents, can shed light on their clinical presentation and help identifying their specific therapeutic needs. Moreover, although indirectly, the high diagnostic stability between past and current ADHD diagnosis found in our study supports the hypothesis of a subtype of BPD with a childhood history of ADHD, hypothesis that has been recently confirmed by Stepp and colleagues in their longitudinal study on adolescent girls [25].

The second limit concerns the small sample size of the study and the potential sample selection bias of the screening phase conducted by the consulting clinicians without performing a systematic between-center inter-rater reliability. This may have reduced the statistical power of the analyses and the generalizability of the results.

For instance, our sample included a majority of female patients. It is commonly agreed that ADHD is less frequent in females, with a predominance of purely inattentional forms. It is possible that the high levels of impulsive features associated with ADHD could be due to a referral bias of our specific clinical sample composed of severe forms of BPD female adolescents. Although the size of the sample of BPD participants was reasonable compared to other studies, particularly since it was limited to adolescents with a well-characterized BPD diagnosis, results should be interpreted with caution as to know what the likelihood might be that the sample is actually representative of BPD adolescents.

Finally, to assess impulsivity, we used the validated adult version of the Barratt Impulsiveness Scale. Although the use of the adult version of the BIS-11 in adolescents can be found in the literature on impulsivity [56,57], it could have been interesting to use the adolescent version of the scale which has been shown to present a different structure from the adult one [58].

## Conclusion

Notwithstanding these limitations, the results of this study confirm, in an adolescent sample, previous studies conducted in adult samples [19] showing that a co-occurring ADHD diagnosis influences the clinical presentation of subjects with borderline personality disorder. ADHD in BPD adolescents was associated with a specific comorbid profile of disruptive disorders, with a trend towards a greater likelihood of cluster B personality disorders, and with higher levels of impulsivity, especially of the Attentional/Cognitive type. These results suggest that BPD in a sub-group of patients could be more developmentally driven, with ADHD symptoms and impairments of the inhibitory system persisting since childhood, thus deserving specific interventions in childhood as well as in adulthood. If confirmed by further empirical evidence, this hypothesis could support the inclusion of a developmental perspective in the DSM classifications of borderline personality disorders. More longitudinal studies are needed to explore the role of these developmental features as risk factors for borderline personality disorders.

## List of abbreviations

ADHD: Attention Deficit Hyperactivity Disorder; BPD: Borderline Personality Disorder; BPD-ADHD: Borderline Personality Disorder with Attention Deficit Hyperactivity Disorder; ODD: Oppositional Defiant Disorder; ASP: Antisocial Personality Disorder.

## Competing interests

The authors declare that they have no competing interests.

## Authors' contributions

All the authors listed in the manuscript have contributed sufficiently to the project to be included as authors. MS intiated and designed the protocol, collected data, participated in data analysis and interpretation and writing and revising the manuscript. ARL participated in data analysis and interpretation and revising the manuscript. SC participated in revising the manuscript. APS and MC participated in designing the protocol, and revising the manuscript. BF participated in data analysis and interpretation and in revising the manuscript. All authors read and approved the final manuscript.

## Pre-publication history

The pre-publication history for this paper can be accessed here:

http://www.biomedcentral.com/1471-244X/11/158/prepub
